# Notes and Notices

**Published:** 1889-02

**Authors:** 


### NOTES AND NOTICES.

Removed.—Dr. Thos. M. Dillingham, from Boston, to 46 West 36th Street, New York City.

Errata.—In Dr. Bell's notice of the Surgical Bureau of the I. H. A., in our January issue, page 39, line 15 from bottom, for *tend* read *attend;* page 40, line 8 from bottom, for *get to know* read *yet know.*

Homœopathic International Congress.—Our French *confrères* desire to have an international gathering of homœopaths at Paris, in August of this year. The French International Exhibition will open in May; many learned societies will convene at Paris during this Exposition. The time is, therefore, appropriate for a gathering of homœopaths. The Secretary of the Commission (of homœopathic doctors having this matter in charge) is Dr. Marc Jousset, 241 Boulevard Saint-Germain, Paris. Any physician who will be able to attend, or any who desire to contribute papers, should notify Dr. Jousset.

Homœopathy again Vindicated.—The Westborough, Mass., Insane Hospital has been open for two years and under homœopathic management. Dr. W. Emmons Paine is the superintendent. A writer in the *Springfield Republican* devotes over a column to showing the good work done in this hospital; he finds the cost of maintenance is much less and the recoveries and general success greater than in allopathic asylums. To the homœopathic system of treatment, and to the diminished use of drugs for sedatives and stimulants, must be ascribed any real increase in the number of recoveries under this system.

